# Response to the letter to the editor: Osteonecrosis of the jaws in patients under osteoporosis treatment: nine-year experience report

**DOI:** 10.20945/2359-4292-2023-0291

**Published:** 2024-03-28

**Authors:** Daniela Cia Penoni, João Vitor S. Canellas, Marcos Antonio Nunes Costa Silami, Flávia Sader, Gonçalo Sobreira Pimentel, Anna Thereza Thomé Leão

**Affiliations:** 1 Hospital Naval de Brasília Divisão de Odontologia Departamento de Saúde Brasília DF Brasil Departamento de Saúde, Divisão de Odontologia, Hospital Naval de Brasília, Marinha do Brasil, Brasília, DF, Brasil; 2 Universidade Federal do Rio de Janeiro Faculdade de Odontologia Departamento de Clínica Odontológica Rio de Janeiro RJ Brasil Departamento de Clínica Odontológica, Divisão de Periodontia, Faculdade de Odontologia, Universidade Federal do Rio de Janeiro, Rio de Janeiro, RJ, Brasil; 3 International Platform of Registered Systematic Review and Meta-analysis Protocols Delaware United States International Platform of Registered Systematic Review and Meta-analysis Protocols, Delaware, United States; 4 Marinha do Brasil Clínica de Estomatologia e Patologia Oral Departamento de Clínica Odontológica Rio de Janeiro RJ Brasil Departamento de Clínica Odontológica, Clínica de Estomatologia e Patologia Oral, Marinha do Brasil, Odontoclínica Central da Marinha, Rio de Janeiro, RJ, Brasil; 5 Marinha do Brasil Divisão de Periodontia Departamento de Clínica Odontológica Rio de Janeiro RJ Brasil Departamento de Clínica Odontológica, Divisão de Periodontia, Marinha do Brasil, Odontoclínica Central da Marinha, Rio de Janeiro, RJ, Brasil; 6 Marinha do Brasil Divisão de Implantodontia Departamento de Clínica Odontológica Rio de Janeiro RJ Brasil Departamento de Clínica Odontológica, Divisão de Implantodontia, Marinha do Brasil, Odontoclínica Central da Marinha, Rio de Janeiro, RJ, Brasil

**DEAR EDITORS AND COLLEAGUES,**

We would like to thank Dr. Efsun Somay for the comments concerning our study (1), which will permit us to clarify some critical points.

Regarding the first comment, "Penoni and cols. did not provide the window period between implant placement and prosthetic loading", we agree that prosthetic loading constitutes an integral aspect of implant therapy. The time required to start the prosthetic loading depends on the surgical technique chosen, which can be immediate implantation, early implantation, or late implantation. This decision is related to the type of implant used, factors inherent to the patient, and the work philosophy of each implant dentistry service, among others (2). Although a good prognosis can be obtained following immediate/early functional or non-functional loading of immediately placed implants, a higher risk of failures seems to exist compared with a delayed, conventional approach (3). In order to provide an estimated window period between implant placement and prosthetic loading in our dental center, we obtained a random sample of 99 placed implants over the 9-year study period using a computer random number generator. A random number table was created using alpha-numeric codes to select 11 patients per year. Of these sample, 75 (75.8%) received late loading (after a complete socket healing); 14 (14.1%) received early loading (after soft tissue healing), and only 10 (10.1%) immediate loading. The late loading is the approach adopted for the majority of our patients. Concerning MRONJ, as properly mentioned in the letter, Escobedo and cols. have stated that using antiresorptive drugs may cause osteonecrosis with implants subjected to functional loading at a higher frequency than after implant placement surgery. After the surgical procedure, peri-implant health over time is essential to prevent MRONJ since the presence and persistence of peri-implant inflammation could trigger it. Based on this knowledge, prostheses and peri-implant health control and maintenance are scheduled regularly in our dental center. This approach, plus the late loading in the great majority of the placed implants, may have contributed to preventing MRONJ over these years.

Regarding the second comment, "the absence of information regarding the duration of antiresorptive drug usage among patients, despite being acknowledged as a limitation of the study, may compromise the accuracy of assessing the impact of antiresorptive use on the incidence of MRONJ." We have already recognized this as a limitation of the study, but some efforts were made to add more information on the duration of antiresorptive drug usage, at least to bring an estimated period. We report data from two studies performed in our dental center during the 9-year period of the present one. These studies involved women with osteoporosis. One revealed a mean period under antiresorptive medication of 4.43 ± 2.72 years in the sample involved (4). The other, a prospective 5-year study recently published, has shown that, among 48 women with osteoporosis, 64.6% were using antiresorptive drugs for more than three years (5). We consider it adequate assuming that a greater part of the procedures performed in the dental center over this 9-year study was in patients who used bone medication for more than four years. So, the higher risk of developing MRONJ for patients who have used bisphosphonates for more than four years has not been absent from the present study.
